# Thermally Robust Non-Wetting Ni-PTFE Electrodeposited Nanocomposite

**DOI:** 10.3390/nano9010002

**Published:** 2018-12-20

**Authors:** Jason Tam, Jonathan Chun Fung Lau, Uwe Erb

**Affiliations:** Department of Materials Science and Engineering, University of Toronto, 184 College Street, Toronto, ON M5S 3E4, Canada; jonathancf.lau@mail.utoronto.ca (J.C.F.L.); uwe.erb@utoronto.ca (U.E.)

**Keywords:** nanocomposite, thermal degradation, surface engineering, non-wettability, electrodeposition

## Abstract

The effect of high temperature exposure on the water wetting properties of co-electrodeposited superhydrophobic nickel-polytetrafluoroethylene (Ni-PTFE) nanocomposite coating on copper substrates was studied. This was accomplished by comparing the performance with a commercial superhydrophobic spray treatment (CSHST). The Ni-PTFE and CSHST coatings were both subjected to heating at temperatures up to 400 °C. Results showed that the Ni-PTFE was able to maintain its superhydrophobicity throughout the entire temperature range, whereas the CSHST became more wettable at 300 °C. Furthermore, additional abrasive wear tests were conducted on both materials that were subjected to heating at 400 °C. The Ni-PTFE remained highly non-wettable even after 60 m of abrasion length on 800 grit silicon carbide paper, whereas the CSHST coating was hydrophilic after 15 m.

## 1. Introduction

Since the late 1990s, there have been large research initiatives worldwide focusing on non-wetting surfaces inspired by various natural structures such as the lotus leaf [1,2,3] or the Nepenthes pitcher plant [4]. In general, non-wetting surfaces can be classified into two types: superhydrophobic surfaces or slippery liquid-infused porous surfaces. In this work, the focus is on superhydrophobic surfaces. These surfaces have a high static water contact angle (WCA) of greater than 150° and a water sliding angle (WSA) of less than 10° [5]. The underlying mechanism for the high repellency of water is a combination of specific surface roughness and low surface energy material [5]. When an external force is applied onto such surfaces, e.g., wind, water droplets will roll off the surface, which facilitates the removal of dirt particles on the surface. This phenomenon is known as the Self-Cleaning Effect. For engineered materials, useful effects arising from surface superhydrophobicity, such as anti-icing, anti-fouling or anti-corrosion have many important applications in different industries, including self-cleaning windows, anti-biofouling boat hulls and corrosion-resistant surfaces. However, there are several challenges for superhydrophobic coatings to be overcome before they are widely adopted. One main problem is that many of their synthesis techniques such as lithography [5,6] chemical vapor deposition [7], or femtosecond laser pulsing [8,9] are usually expensive and not easily scalable for large scale commercial applications. Another problem is that many superhydrophobic products that utilize low surface energy surface treatments such as spray coatings or paints are easily degraded when worn, so they do not provide long lasting hydrophobic effects for the coated material [10,11]. Therefore, there is a need for durable and long lasting, yet low-cost superhydrophobic surface treatments/coatings. Durability and robustness of a surface does not only refer to physical degradation, for example through mechanical wear, but also thermal degradation such as through intentional or unintentional temperature excursions during service.

The topic of physical wear of superhydrophobic materials has been studied extensively across a variety of materials, including polymers, metals and metal oxides functionalized with low surface energy coating, and composites [11,12,13,14,15,16,17,18,19,20,21,22,23,24,25,26,27,28,29,30], as it is one of the main concerns with their durability. Among these studies, only a subset evaluated the thermal stability in addition to abrasive wear resistance [25,26,27,28]. For instance, superhydrophobic TiO_2_ coatings modified with stearic acid were able to withstand sand abrasion and possessed thermal stability where superhydrophobic properties were retained after heat treatment at 200 °C [26]. There are few other studies that reported thermal stability of superhydrophobic surfaces [31,32,33,34], including nanostructured Si coated with hydrophobic diamond-like carbon [32] and polyphenylsilsesquioxane/nanosilica composites [34], which were thermally stable up to 400 °C and 500 °C, respectively. However, these studies did not assess the abrasive wear resistance of the surfaces. Currently, there are no standards in evaluating the thermal stability of superhydrophobic surfaces. In most of the previous studies, thermal stability of non-wetting surfaces was assessed by exposing the specimens in air to elevated temperatures for 10 min [31], 30 min [26,27], 1 h [25,28,33], or 24 h [32]. WCA measurements were conducted after the specimen cooled down to ambient temperature.

**Figure 1:**
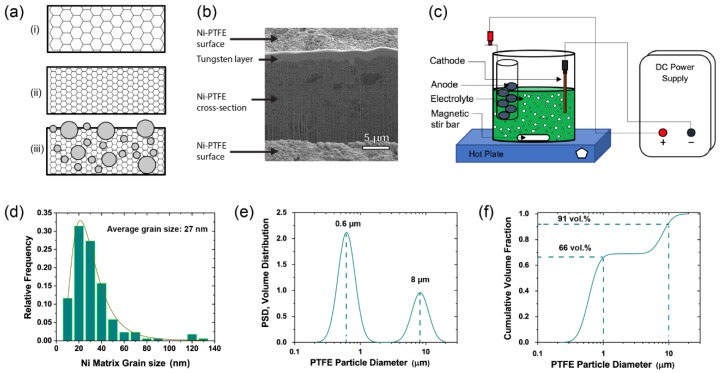
(**a**) Cross-sectional schematic diagrams of various electrodeposits: (**i**). Polycrystalline metal, (**ii**). Nanocrystalline metal, (**iii**). Nanocomposite with nanocrystalline metal matrix and co-deposited hydrophobic particles. (**b**) Secondary electron image of Ni-PTFE cross-section after FIB milling. (**c**) Schematic diagram of electroplating setup. (**d**) Grain size distribution of nanocrystalline nickel matrix. (**e**) Particle size distribution and (**f**) cumulative volume fraction of PTFE particles.

The basic idea of making a conceptually different wear resistant superhydrophobic nanocomposite surface using a simple electrochemical approach has been described in our previous studies [10,17,35]. This method uses electro-codeposition, a technique that has been extensively applied in the past to fabricate nanocomposites to achieve improved surface properties of a coated surface [36,37,38,39], for example higher strength and hardness by co-depositing nano-sized SiC particles with nickel [40]. The superhydrophobic nanocomposite surfaces reported in this study were fabricated by electro-codeposition of a fully dense nanocrystalline metal along with embedded hydrophobic particles throughout the coating. The dual-scale surface roughness of this surface mimics superhydrophobic surfaces found in nature [1,41]. Firstly, by refining the grain size of the deposited metal from conventional polycrystalline as seen in Figure 1a(i), to nanocrystalline shown in Figure 1a(ii), the electrodeposited metal will follow the Hall-Petch relationship [42,43] where the decreasing grain size will contribute to higher strength and hardness, and therefore enhanced wear resistance [44]. Next, by co-depositing hydrophobic second phase particles uniformly throughout the thickness of the coating as shown in Figure 1a(iii), the surface continually allows new hydrophobic particles (e.g., PTFE, CeO_2_) to be exposed to the surface of the material even when the original surface is worn away. This approach results in a long lasting and mechanically robust superhydrophobic surface [11,17]. In a sense this is similar to the self-repair capability previously observed on some tree leaves which show a continuous regrowth throughout the growing season of worn off wax crystals on top of papillae responsible for superhydrophobicity [45].

In this work, we evaluated the thermal stability of superhydrophobicity of Ni-PTFE nanocomposite and compared the results with a CSHST. It is shown that Ni-PTFE nanocomposites fabricated by the electrodeposition possess high thermal stability with no degradation of the non-wetting properties even after heat treatment up to 400 °C. In addition, unlike previous studies, we evaluated the adhesion of the Ni-PTFE nanocomposites to the substrate by a cross-cut tape test and the wear resistance by an abrasion test after they had been subjected to heat treatment at 400 °C.

## 2. Materials and Methods

The non-wetting material studied in this research is a Ni-PTFE nanocomposite coating prepared through the aforementioned electrodeposition procedure developed earlier by Iacovetta et al. [10]. The electrodeposition was carried out in 400 mL beakers with a modified Watts plating bath containing 300 g/L NiSO_4_·6H_2_O, 45 g/L NiCl_2_·6H_2_O, 45 g/L boric acid, and 30 g/L PTFE particles. The as-deposited composite contained about 70 vol% PTFE on the surface. Inco nickel rounds contained in a titanium mesh basket were used as the dissolvable anode. The cathode substrates used were 2 cm × 2 cm polished copper coupons. The current density used was 100 mA/cm^2^, with an electroplating bath temperature of 60 °C and a plating time of 15 min. The Ni-PTFE composite coatings were electrodeposited to a thickness of about 60 µm. After electrodeposition, the specimens were rinsed with deionized water, followed by ultrasonic cleaning in ethanol. The electrodeposition setup can be seen in Figure 1c. The electrodeposited nickel matrix has an average grain size of 27 nm and follows a logarithmic normal distribution as shown in Figure 1d. The electrodeposited composite contains PTFE particles with a bimodal particle size distribution (PSD) as shown in Figure 1e. The micrometer and submicrometer sized particles had average particle sizes of 8 µm and 0.6 µm, respectively. As shown in Figure 1f, the volume fraction of the PTFE particles having a particle size less than 1 µm was about 66%. This bimodal distribution is essential in the durability of the superhydrophobic surface. In our previous study [11], we reported that owing to the dual-scale roughness of the Ni-PTFE nanocomposite coating, the surface was able to sustain long abrasion lengths and displayed high mechanical and non-wetting durability through the continuous exposure of new hydrophobic particles over the thickness of the coating with increasing wear. In Figure 1b, a secondary electron image of a cross-section of a Ni-PTFE sample is shown. The cross-section was made by using focused ion beam (FIB) milling to observe the particle distribution throughout the thickness of the coating. As seen in the figure, there is an even distribution of PTFE particles throughout the coating allowing new particles to be exposed as the surfaces gets worn down and thus, having a robust and durable superhydrophobic coating.

The other superhydrophobic material used here to compare and contrast with the Ni-PTFE composite was a commercial superhydrophobic spray treatment (CSHST): NeverWet™. It was also applied on 2 cm × 2 cm polished copper coupons according to the manufacturer’s instructions.

To investigate the thermal degradation for Ni-PTFE and CSHST samples, they were both subjected to heating in a box furnace. The samples were placed in an alumina crucible and wrapped in aluminum foil to minimize oxidation. Both the Ni-PTFE and CSHST samples were heated together at temperatures of 150 °C, 200 °C, 250 °C, 300 °C, 350 °C, and 400 °C for 1 h each. After heating, time was allowed for the furnace to cool down to room temperature (25 °C) before samples were removed. Their WCA’s were then measured using 5 µL droplets of deionized water. Samples were also examined with a Hitachi SU5000 scanning electron microscope (SEM) (Hitachi, Tokyo, Japan) to observe any changes in their surface morphologies after heating that may affect wettability of the surfaces. In addition, differential scanning calorimetry (DSC) of the PTFE particles used to produce the Ni-PTFE nanocomposites was performed with a Netzsch STA 449 F3 Jupiter simultaneous thermal analyzer (Netzsch, Selb, Germany). The experiment was carried out in a nitrogen atmosphere and the specimen was heated from 40 °C to 730 °C at a heating rate of 10 °C/min.

## 3. Results and Discussion

### 3.1. Characterization of As-Deposited Materials

**Figure 2:**
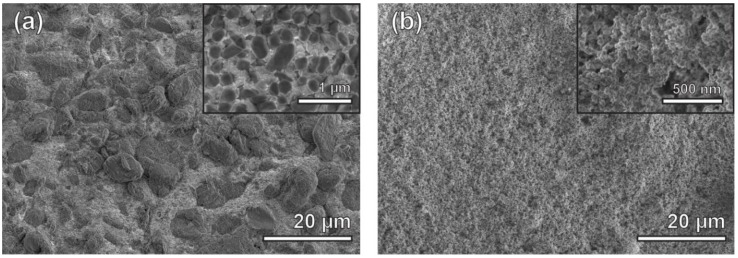
Secondary electron micrographs of (**a**) as-deposited Ni-70 vol% PTFE with dual scale roughness, inset showing embedded submicron size PTFE particles in nanocrystalline Ni matrix. (**b**) As-deposited CSHST, inset showing the fine nanoscale roughness and porous structure.

**Figure 3:**
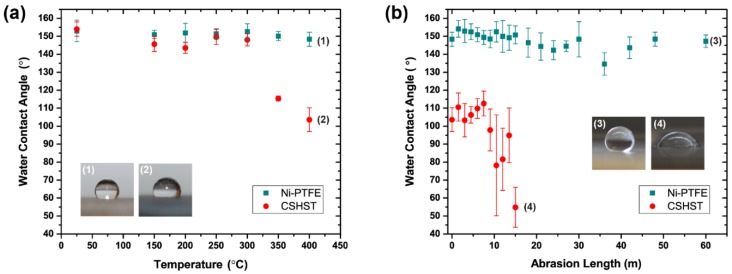
(**a**) WCA (Water Contact Angle) of Ni-70 vol% PTFE and CSHST after 60 min of heating at various temperatures. Error bars show the standard deviation of the measurements. Insets 1 and 2 show macro photographs of water droplets on Ni-PTFE and CSHST respectively after the 400 °C heat treatment. (**b**) WCA of Ni-PTFE and CSHST after 60 min of heating at 400 °C and subjected to abrasive wear on 800 grit silicon carbide paper. Insets 3 and 4 show macro photographs of water droplets on Ni-PTFE and CSHST after heating to 400 °C and after abrasive wear on 800 grit silicon carbide paper over abrasion lengths of 60 m and 15 m, respectively.

Secondary electron micrographs of as-deposited Ni-PTFE and CSHST are shown in Figure 2a,b, respectively. The Ni-PTFE micrographs show dual scale roughness with large micron sized PTFE particles, as well as submicron sized PTFE particles as shown more closely in the inset. This reflects the bimodal distribution of 8 µm and 0.6 µm particles added to the plating bath, Figure 1c,e. In contrast, the as-deposited CSHST displays a porous structure with fine nanoscale roughness. While the exact composition of CSHST is proprietary, according to the material safety data sheet, the CSHST contains silicone rubber and silica [46]. Based on these data, it can be hypothesized that the CSHST is composed of SiO_2_ nanoparticles with a hydrophobic silicone polymer binder to keep the particles together. Both as-deposited materials are superhydrophobic with WCAs > 150° (Figure 3a).

### 3.2. High Temperature Exposure

**Figure 4:**
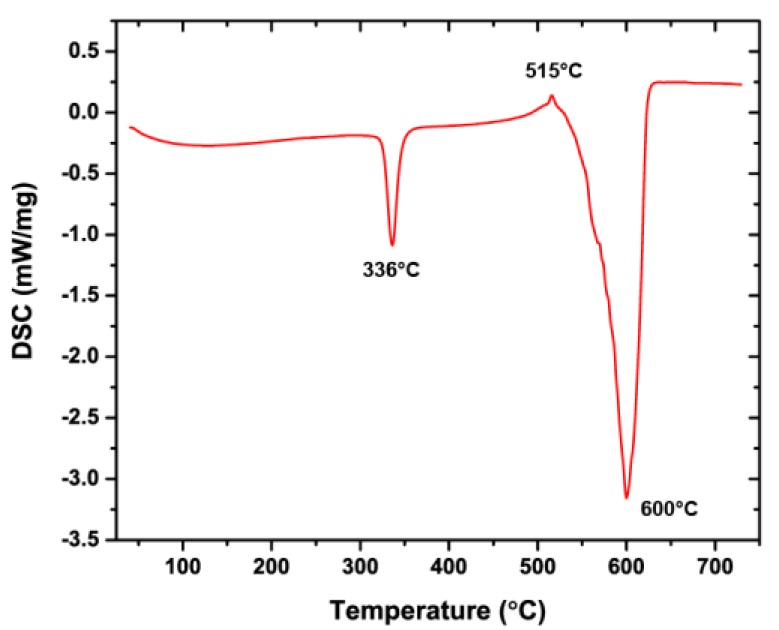
DSC curve of PTFE powder. The melting point of PTFE is 336 °C and decomposition initiates at 515 °C.

The wetting angle measurements after heat treating the materials are presented in Figure 3a. After being subjected to 60 min of heating at 150 °C, 200 °C, 250 °C, 300 °C, 350 °C and 400 °C, the Ni-PTFE samples did not exhibit a major decrease in WCA and were able to maintain their superhydrophobicity. This result is expected as PTFE does not decompose until the temperature is greater than 515 °C [47], as shown in the differential scanning calorimetry curve of the PTFE powder (Figure 4). In contrast, the non-wettability of the CSHST samples started to decrease significantly at 350 °C with a contact angle of just 104° after annealing at 400 °C.

**Figure 5:**
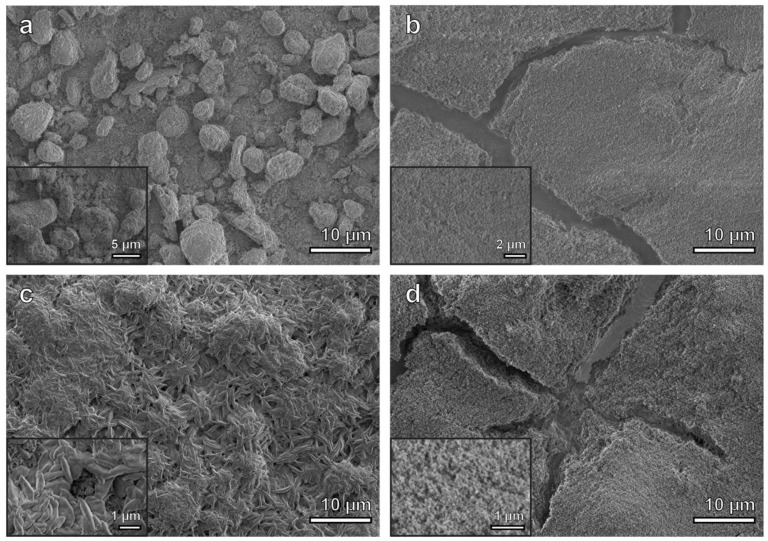
Secondary electron micrographs of (**a**) Ni-70 vol% PTFE sample after 200 °C of heating, inset shows higher magnification image with individual PTFE particles. (**b**) CSHST sample after 200 °C of heating, inset shows higher magnification image of the nanoscale roughness on the surface. (**c**) Ni-70 vol% PTFE sample after 350 °C of heating, inset shows higher magnification image of the strand-like structure dispersed over the surface of the sample. (**d**) CSHST sample after 350 °C of heating, inset shows higher magnification image of the nanoscale roughness on the surface.

Secondary electron micrographs of the Ni-PTFE and CSHST coatings after heating at 200 °C are shown in Figure 5a,b, respectively. The surface structure of the Ni-PTFE still shows a dual scale roughness with a distribution of small and large PTFE particle agglomerates. There are no significant changes from the as-deposited Ni-PTFE structure shown in Figure 2. This is expected, as the melting point of PTFE is 336 °C (Figure 4). Thus, the Ni-PTFE coating was able to maintain its superhydrophobicity. In contrast, after annealing at 200 °C, the surface of the CSHST looks quite different (Figure 5b) from the as-deposited state (Figure 2b). Although the original nanoscale roughness of the coating is still present, surface cracks can be seen throughout the CSHST sample. However, even with the presence of these cracks on the surface, CSHST maintained superhydrophobicity after heating to 200 °C (Figure 3a). This could be attributed to the size of the cracks, as they are still narrow relative to the size of water droplet.

The most likely reason that cracks were observed after the heating/cooling cycle in the CSHST coating but not in the Ni-PTFE coating is the difference in the coefficient of thermal expansion (CTE) of the coating materials and the copper substrate: CTE of copper = 17 × 10^−6^ K^−1^ [48], CTE of nickel = 13 × 10^−6^ K^−1^ [48], CTE of PTFE = 100 × 10^−6^ K^−1^ [49], CTE of silica = 0.6 × 10^−6^ K^−1^ [49], CTE of silicone rubber = 300 × 10^−6^ K^−1^ [50]. For both constituents of the CSHST coating, the CTE is at least one order of magnitude larger/smaller than for the copper substrate. On the other hand, for the Ni-PTFE composite, at least the CTE of Ni matrix is closely matched to the copper substrate and the compressibility of PTFE is very high [51]. This demonstrates that, for applications of any superhydrophobic coating which could be subjected to high temperature excursions during service, the thermal mismatch between the coating and the substrate should be taken into consideration.

Secondary electron micrographs of the Ni-PTFE and CSHST after heating to 350 °C are shown in Figure 5c,d, respectively. A Ni-PTFE coating with quite a different surface structure can be seen. The PTFE particles have melted and coalesced to a strand-like structure after being heated to 350 °C. Smaller round shaped PTFE particles are no longer present. It is important to note that most of the surface of the coating is now covered with these newly solidified elongated PTFE particles. The elongated morphology is consistent with a previous study that investigated the crystallization of dispersed PTFE particles [52]. Although the shape of the PTFE has changed, hierarchical surface roughness is still apparent. Larger microscale agglomerates of elongated PTFE particles in addition to the smaller submicrometer wide elongated PTFE particles contribute to the dual scale roughness of the composite, allowing the Ni-PTFE coating to maintain superhydrophobicity (Figure 3a). On the contrary, an extensive network of cracks is visible on the CSHST surface. The cracks on the surface are much more pronounced than at 200 °C and cover a larger area fraction. This could be the reason why the superhydrophobicity of the CSHST was not maintained at 350 °C (Figure 3a). As previously reported, partial exposure to the hydrophilic copper substrate can contribute greatly to the decreased wettability of the CSHST coating [11].

**Figure 6:**
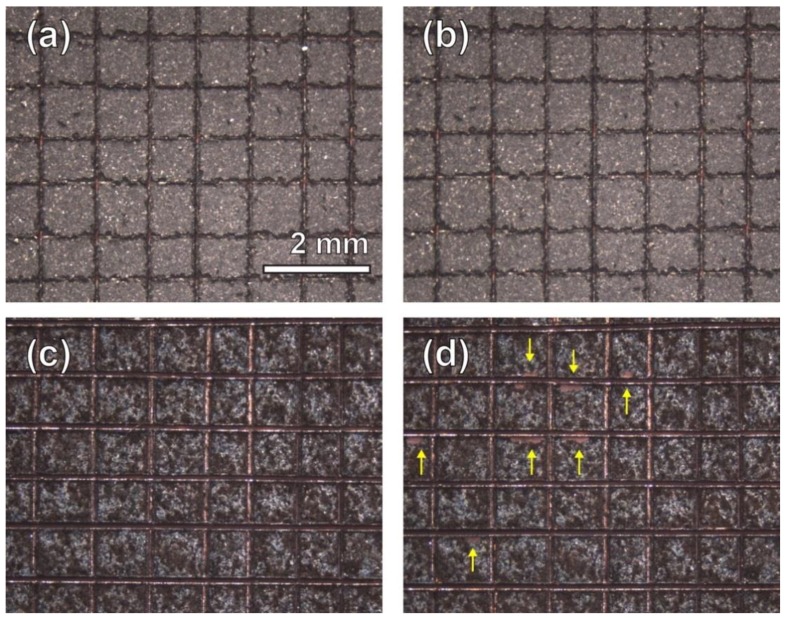
Cross-cut tape test of superhydrophobic coatings after heating to 400 °C for 60 min. (**a**,**b**) Ni-70 vol% PTFE before and after the tape test. None of the squares of the lattice were detached. (**c**,**d**) CSHST coatings before and after the tape test. The arrows indicate regions of coating delamination.

Adhesion of the coatings on the substrate after heating to 400 °C was evaluated by a cross-cut tape test according to ASTM D3359-09 [53] (Figure 6). No removal of lattice squares was observed for the Ni-PTFE nanocomposite coating (Figure 6b). However, some regions of the CSHST coating were delaminated after the tape test. Based on the rating scale of ASTM D3359-09, classification of 5B (no detachment of the squares of the lattice) and 3B (small flakes of the coating are detached along edges and at intersections of cuts) can be assigned for Ni-PTFE and CSHST, respectively.

**Figure 7:**
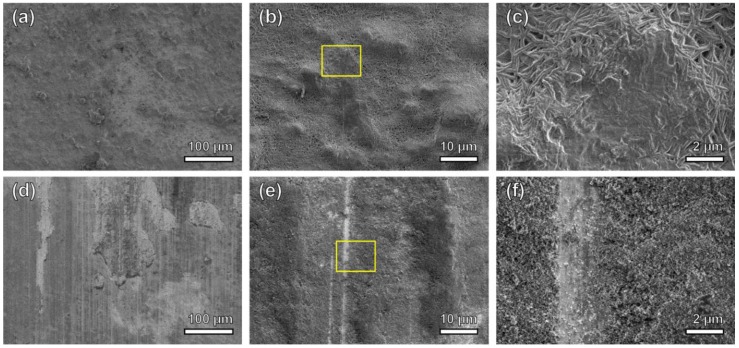
Secondary electron micrographs of both coatings after heating at 400 °C for 1 h, followed by 60 m of abrasion for Ni-70 vol% PTFE (**a**–**c**) and 15 m of abrasion for CSHST (**d**–**f**). (**a**) Low magnification, (**b**) medium magnification, showing the micro scale, protruding PTFE agglomerates were smoothened after abrasion. (**c**) High magnification of the region marked by the box in (**b**); the submicron-scale PTFE strands superimposed on the microscale protrusions were slightly damaged by the abrasion again 800 grit SiC, while the lower lying features remained intact. In comparison, the CSHST experienced severe damage after heating at 400 °C for 1 h, followed by 15 m of abrasion (**d**–**f**). The low magnification image (**d**) shows that significant delamination of the coating occurred. In addition, the medium magnification image (**e**) demonstrates that the surface morphology changed significantly at the microscale. The region marked by the box is shown as a high magnification image (**f**), where micron size wear tracks generated from the abrasion can be observed.

To investigate the wear resistance of the coating after high temperature exposure, a wear test was performed using the same parameters that were used to test the same materials in our previous study for the as-deposited state [11]. Using a simple abrasion wear test apparatus, 2000 Pa of downward force was applied to both the Ni-PTFE and CSHST samples after heating to 400 °C o ver an abrasion length of up to 60 m using 800 grit silicon carbide paper. The same Ni-PTFE and CSHST samples were used as the ones labeled as points 1 and 2 in Figure 3a. A summary of the results can be seen in Figure 3b. The Ni-PTFE sample was able to retain WCAs close to 150° for up to at least 60 m without major loss of the highly non-wetting properties. Secondary electron images after the abrasion test are shown in Figure 7a–c; some micro scale roughness can still be observed on the low magnification image (Figure 7a). At higher magnifications (Figure 7b,c), it can be seen that the PTFE strands superimposed on the microscale protrusions were destroyed by abrasion, while the lower lying submicron PTFE strands remained intact, still allowing remarkable non-wetting properties even after annealing and abrasion. In contrast, CSHST coatings annealed at 400 °C are only slightly hydrophobic (104°, Figure 3a) and became hydrophilic after relatively short abrasion exposure, mainly due to the pre-existing microcracks formed at a lower heating temperature (Figure 5d) that accelerated the delamination of the coating from the substrate during abrasion and the exposed hydrophilic Cu substrate can be clearly observed (Figure 7d–f).

Currently the Ni-PTFE coating system described in this communication is being scaled up for larger and more complex shaped substrates of industrial relevance. This will also include an assessment of the coating performance after longer high temperature exposure.

## 4. Conclusions

This study demonstrated the thermal degradation resistance of electrodeposited Ni-PTFE nanocomposite coatings in comparison to a CSHST. Both coatings were subjected to 1-h heat treatments at temperatures of 150 °C, 200 °C, 250 °C, 300 °C, 350 °C and 400 °C, respectively. The results showed that the Ni-PTFE coating was able to maintain superhydrophobicity even after heating to 400 °C, whereas the CSHST started losing its non-wetting behavior at temperatures above 300 °C. After exposure to 400 °C, both types of specimens were also subjected to an abrasive wear test to investigate their wear resistance after elevated temperature excursions. Results showed that the Ni-PTFE coating was able to maintain superhydrophobicity after 60 m of abrasion length on 800 grit silicon carbide paper. However, the CSHST failed to even maintain hydrophobicity after 15 m of abrasion. This study demonstrated the robustness of Ni-PTFE composites; they can tolerate higher temperature excursions and are mechanically more robust than the CSHST. The results of this study further demonstrate the applicability of robust, non-wetting Ni-PTFE nanocomposite coatings in industrial settings where the surface can be exposed to intentional/unintentional thermal excursions as well as abrasive wear.
